# A Highly Active Chondroitin Sulfate Lyase ABC for Enzymatic Depolymerization of Chondroitin Sulfate

**DOI:** 10.3390/polym14091770

**Published:** 2022-04-27

**Authors:** Xiao-Man Fan, Jia-Ying Huang, Xiao-Min Ling, Wei Wei, Wen-Bin Su, Ye-Wang Zhang

**Affiliations:** School of Pharmacy, Jiangsu University, Zhenjiang 212013, China; fanxiaomanmail@yeah.net (X.-M.F.); jiayinghuang98@yeah.net (J.-Y.H.); 18260627002@163.com (X.-M.L.); zhongshiweiwei@163.com (W.W.); 3141601022@stmail.ujs.edu.cn (W.-B.S.)

**Keywords:** *Bacteroides thetaiotaomicron*, high activity, characterization, depolymerization, low-molecular-weight chondroitin sulfate

## Abstract

Enzymatic preparation of low-molecular-weight chondroitin sulfate (LMWCS) has received increasing attention. In this work, a chondroitin sulfate lyase ABC (Chon-ABC) was successfully cloned, expressed, and characterized. The *K_m_* and *V_max_* of the Chon-ABC were 0.54 mM and 541.3 U mg^−1^, respectively. The maximal activity was assayed as 500.4 U mg^−1^ at 37 °C in pH 8.0 phosphate buffer saline. The half-lives of the Chon-ABC were 133 d and 127 min at 4 °C and 37 °C, respectively. Enzymatic preparation of LMWCS was performed at room temperature for 30 min. The changes between the substrate and product were analyzed with mass spectrometry (MS), high-performance liquid chromatography (HPLC), gel permeation chromatography (GPC), and nuclear magnetic resonance (NMR). Overall, the Chon-ABC from *Bacteroides thetaiotaomicron* is competitive in large-scale enzymatic preparation of LMWCS for its high activity, stability, and substrate specificity.

## 1. Introduction

Chondroitin sulfate (CS) is an anionic bio-macromolecule of glycosaminoglycans (GAGs), which usually covalently link to proteins [1]. It plays important roles in many biological processes, such as neuronal development, coagulation, joint inflammation, cellular trafficking, and proliferation [2,3,4,5,6]. Moreover, it has been used as a food preservative with emulsifying features [7], as well as for preventing and reducing osteoarthritis by administering in nutraceutical formulations [8]. Even though CS extracted from diffident cartilage tissues has been used as medicine [9] or dietary supplements [10], recent reports demonstrated that low-molecular-weight chondroitin sulfate (LMWCS) is more bioactive and readily absorbed by gastrointestinal mucosa [11,12,13]. LMWCS is generally obtained by degrading CS, which is composed of several repeat disaccharide units connected via a β-(1→4) glycosidic bond. Its molecular weight is about 3000–8000 Da, containing unsaturated disaccharides produced during degradation. To prepare LMWCS, the enzymatic method has the advantages of mild conditions and high substrate specificity compared with the conventional ways such as acidic, oxidative, and radiation methods [6,14,15,16]. For example, acid methods through nitrous acid would deammonify CS oligosaccharides and produce 2,5-anhydromannose at the reducing terminal, and multiple side reactions would occur in degradation leading to difficulty in further isolation and purification, as well as the problem of low yield [17,18]. LMWCS produced from the enzymatic conversion of CS with chondroitin lyase has better biological activity, and in this process, uronic acid could be specifically converted into 4,5-unsaturated acid. Therefore, the enzymatic method is promising in producing LMWCS [19,20].

Chondroitin sulfate ABC lyase (Chon-ABC, EC 4.2.2.4) is a member of glycosaminidases with broad substrate specificity, which can degrade CS glycosaminoglycans (GAGs) through β-elimination reaction [21]. It is capable of depolymerizing CS into Δ4,5-unsaturated LMWCS, which has the potential to be used in therapeutic and industrial fields [22,23,24]. Up to now, different Chon-ABCs from *Proteus vulgaris* [25,26,27], *Bacteroides thetaiotaomicron* [28], *Bacteroides stercoris* [29], and *Acinetobacter sp*. [30] have been identified and characterized.

Many Chon-ABCs from different species were reported to be prepared through fermentation and purification, while the drawbacks of low enzyme activity and complicated extraction processes hamper their further applications in larger-scale production. To decrease the cost, heterologous expression is an efficient strategy [25,31,32,33]. However, most Chon-ABCs still cannot meet the industrial requirements owing to the low expression and specific activity. Li et al. [34] introduced molecular chaperones and heterologously expressed Chon-ABC to increase the soluble expression and activity, and the results suggest that the tedious purification process of Chon-ABC and high cost were the main obstacles.

In the present work, an efficient heterologous expression of a Chon-ABC from *B. thetaiotaomicron* was successfully conducted. The purified enzyme was characterized, and the evaluation of possible applications in depolymerization of CS was performed. The substrate and the enzymatic hydrolyzed CS were analyzed by gel permeation chromatography (GPC), high-performance liquid chromatography (HPLC), mass spectrometry (MS), and nuclear magnetic resonance (NMR) to explore the molecular weight and structural changes after enzymatic depolymerization.

## 2. Materials and Methods

### 2.1. Bacterial Strains, Plasmids and Chemicals

*B. thetaiotaomicron* was obtained from the Guangdong culture collection center (Guangzhou, China). Strain *Escherichia coli* Top10 and BL21 (DE3) pLysS were purchased from Sangon Biotech (Shanghai, China). The SanPrep Column Plasmid Mini-Preps Kit was from Sangon Biotech (Shanghai, China). The plasmid pCzn1 was ordered from Zoonbio Biotechnology Co., Ltd. (Shanghai, China). Chondroitin sulfate of shark cartilage was ordered from Sigma-Aldrich (Shanghai, China). Isopropyl-beta-D-thiogalactopyranoside (IPTG) was purchased from Energy Chemical (Shanghai, China). Other reagents used in the experiment were all analytic grade and supplied by Sinopharm Chemical Reagent Co., Ltd. (Shanghai, China).

### 2.2. Cloning and Expression of the Recombinant Chon-ABC

The genomic DNA from *B. thetaiotaomicron* was extracted through the SanPrep column plasmid mini-preps kit. Then polymerase chain reaction (PCR) was carried out to amplify the gene with DNA polymerase and the designed primers containing restriction sites for *Nde*I and *Xba*I (the sequences were: F 5′-CATATGCAGGTCATAGGGTTTGAAG-3′ and R 5′-AGATCTTTTATTTTCAAGAATCATTTC-3′). The purified 2.8 kb PCR fragments were ligated into the vector pCznl to construct the recombinant plasmid pCznl-Chon-ABC. Then the pCznl-Chon-ABC was transformed into the competent cell of *E. coli* BL21 DE3 (pLysS) to express the target protein. All the constructed recombinant plasmids were confirmed by DNA sequencing.

Several single colonies of *E. coli* BL21 (DE3) pLysS, which carried the recombinant pCzn1-chon-ABC, were picked from the plate and incubated to a flask loading 1 L of LB culture medium (10 g L^−1^ NaCl, 10 g L^−1^ tryptone, and 5 g L^−1^ yeast extract) containing 50 μg mL^−1^ ampicillin. The cells were cultured at 220 rpm, 37 °C for 8–10 h. When the OD_600_ reached 0.6–0.8, they were induced by 0.75 mM IPTG and cultivated for another 6 h at 25 °C to overexpress the Chon-ABC.

For the extraction of intracellular proteins, the cells were centrifuged 10 min at 1641× *g* at 4 °C and washed twice with a washing solution (pH 8.0 200 mM phosphate buffer saline (PBS, consisting of Na_2_HPO_4_ and KH_2_PO_4_), 250 mM imidazole, and 150 mM NaCl). Then the collected cells were resuspended in the washing solution and disrupted by ultrasonication. After removing the debris with 20 min centrifugation at 6484× *g*, the supernatant was poured into the nickel affinity chromatography column (Beyotime Bio, Shanghai, China), which was pre-treated with a binding buffer (pH 8.0 20 mM PBS, containing 150 mM NaCl). Proteins without His-tag were rinsed out of the column through the washing buffer (pH 8.0 20 mM PBS, containing 50 mM imidazole and 150 mM NaCl). The recombinant Chon-ABC was obtained from the column with an elution buffer (pH 8.0 20 mM PBS, containing 250 mM imidazole and 150 mM NaCl). All the samples were analyzed with sodium dodecyl sulfate–polyacrylamide gel electrophoresis (SDS-PAGE) using 12% gradient gel.

### 2.3. Enzyme Assay

The enzyme activity of Chon-ABC was calculated by measuring the formation of unsaturated uronic acid at 232 nm during the enzymatic degradation of the polysaccharide [35]. Briefly, 5 μL enzyme solution was mixed with 995 μL 75 mM PBS (pH 8.0) containing 1 mg mL^−1^ CS at 37 °C. The calculation was performed through the change of adsorption at 232 nm in an ultraviolet spectrophotometer. The molar extinction coefficient of unsaturated uronic acid is 5100 L (mole cm)^−1^. One international unit was defined as the amount of the enzyme which can produce one micromole unsaturated uronic acid per minute.

### 2.4. Biochemical Characterization of the Recombinant Chon-ABC

The effect of temperature on the activity of the recombinant Chon-ABC was investigated over the range of 20–60 °C in 75 mM phosphate saline (pH 8.0). The buffer was preheated at the designed temperatures, then the enzyme was added to evaluate the relationship between the enzyme activity and temperature. The effect of pH on the Chon-ABC activity was determined at 37 °C in 50 mM citrate buffer (pH 4.0–6.0), PBS (pH 6.0–8.0), and Tris-HCl (pH 8.0–9.0), respectively. The effect of buffer concentration on the enzyme activity was determined using various concentrations of PBS from 10 to 250 mM at pH 8.0. The effects of metal ions and surfactants on the Chon-ABC activity were assessed under the conditions of 1 mM metal ions (Mn^2+^, Co^2+^, Mg^2+^, Ca^2+^, Li^+^), 1 mM EDTA, and 5% surfactant (tween 80), respectively. The reaction solution without surfactants and metal ions was set as a control group. The standard deviation was calculated from the three recorded experimental data for each experiment.

### 2.5. Thermal and Storage Stabilities of the Chon-ABC

The thermostability of the Chon-ABC was examined by incubating the enzyme at 37 °C for 2–120 min. The storage stability of the Chon-ABC was detected at 4 °C for 107 days. The half-life ($t_{\frac{1}{2}}$) of the recombinant Chon-ABC was calculated with the following equation:

$$t_{\frac{1}{2}}=\frac{0.693}{k_{d}}$$

where *k_d_* represents the elimination rate constant which is calculated by the formula:

$$k_{d}=\frac{\ln\frac{E_{0}}{E_{t}}}{t}$$

where *E*_0_ means the initial enzyme activity, and *E_t_* represents the enzyme activity incubated after t min.

### 2.6. Kinetic Parameters of the Recombinant Chon-ABC

The *V_max_* and *K_m_* values of the recombinant Chon-ABC were measured and calculated with CS solutions from 0 to 3 mg mL^−1^. Typically, 5 μL enzyme solution was mixed with 995 μL different concentrations of CS solution, then the absorbance change was recorded in 232 nm. The kinetic parameters were obtained from the non-line regression fitting (Michaelis–Menten). All assays were repeated three times, and the standard deviation of each statistically relevant data was less than 10%.

### 2.7. Enzymatic Depolymerization of CS

Enzymatic preparation of LMWCS was performed in a 250 mL shake flask, and 250 mg CS was mixed with 50 mL purified enzyme solution (3 U mL^−1^), then the mixture was incubated at 37 °C and 220 rpm for 30 min. Thereafter, the solution was boiled for 10 min to terminate the reaction. When the mixture was cooled to room temperature, the supernatant was obtained by centrifugation at 6484× *g* for 20 min.

### 2.8. Analysis of CS and the Enzymatic Depolymerized Products

Strong anion-exchange HPLC and GPC were used to detect LMWCS production. The product after hydrolysis was analyzed by MS. HPLC was performed by a ZORBAX SAX column (Agilent, 4.6 × 250 mm, 5 μm) at 40 °C. Eluents A and B were 2 M NaCl (pH 3.5) and ultrapure water (pH 3.5), respectively [36]. Gradient separation was performed using eluent A followed by a linear gradient from 0 to 1 M at a flow rate of 0.75 mL min^−1^ for 45 min. GPC was performed by an Ultrahydrogel^TM^ Linear 7.8 × 300 mm column at 40 °C using 0.1 M NaNO_3_ as the eluent at the rate of 0.5 mL min^−1^. The hydrolysate of CS was analyzed by ESI-MS (Thermo, Waltham, MA, USA) in negative ion mode. The mass analysis was performed for 15 min. The negative ion mode was used with the sheath gas flow rate at 20 arb and aux gas flow rate at 5 arb; the spray voltage was 3.5 KV; the capillary temperature was 275 °C; the capillary voltage was −40 V; and the tube lens was −50 V. In addition, the scan range of positron ionization was 100–2000 *m/z*. The MS analysis was offline.

Moreover, NMR was performed to analyze the structural changes of the CS and hydrolyzed CS. The freeze-dried sample (25 mg) of CS and enzymatic hydrolyzed CS was dissolved in 1 mL deuterium oxide. ^1^H NMR and ^13^C NMR of the CS and hydrolyzed CS were obtained by using an Agilent DD2-600 spectrometer (Agilent Technologies, Santa Clara, CA, USA) at 25 °C with 600 MHz nuclear magnetic frequency.

### 2.9. Molecular Docking of the Chon-ABC and Sulfated Glycosaminoglycan

Molecular docking of the Chon-ABC with sulfated glycosaminoglycan was performed by AutoDock Tools [37]. The tertiary structure of Chon-ABC was generated with the Swiss-model online server (https://swissmodel.expasy.org/, accessed on 4 December 2021) using the Chon-ABC from proteus vulgaris (PDB: 1HN0) as the templet. The structure of the sulfated glycosaminoglycan was downloaded from the PubChem online database (https://pubchem.ncbi.nlm.nih.gov/, accessed on 4 December 2021). The structure of the chondroitinase ABC (PDB: 1HN0) was downloaded from the PDB database (http://www.rcsb.org/, accessed on 4 December 2021). The molecular docking was performed by AutoDock Tools with the default parameters, of which the grid space was 0.735 Å, and the number of runs was 100 in Lamarckian genetic algorithm. The free energy of the complex was calculated using Gromacs-5.1 [38].

## 3. Results

### 3.1. Cloning, Expression, and Purification of the Chon-ABC

**Figure 1:**
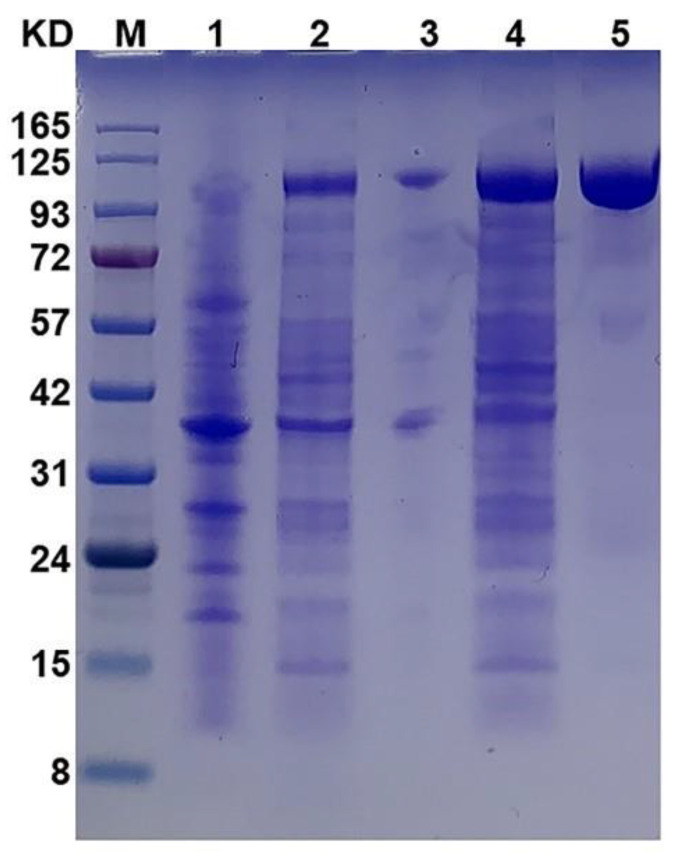
SDS-PAGE of Chon-ABC with 4–12% gradient. M: protein marker; Lane 1: cell lysate before induction; Lane 2: cell lysate after induction with IPTG; Lane 3: the supernatant after sonification; Lane 4: the precipitate after sonification; Lane 5: the purified Chon-ABC by Ni-NTA column.

The gene of the Chon-ABC was cloned from *B. thetaiotaomicron* with 2862 bp. It encoded 953 amino acids with a molecular weight of 108.3 kDa and an isoelectric point of 7.64. The Chon-ABC was successfully expressed in *E. coli* BL21 DE3 (pLysS) with a 6 × His-tag at the terminal. After cell culture, induction, and ultrasonication, the total protein extracted from *E. coli* was analyzed by 12% SDS-PAGE. After purification with the Ni-NTA column, a single band with a molecular weight of approximately 108 kDa protein was observed in SDS-PAGE (Figure 1), which agrees with the prediction. The purified Chon-ABC showed high activity toward CS. These results demonstrate that the molecular weight of the enzyme expressed by the *E. coli* BL21 DE3 (pLysS) is consistent with the putative Chon-ABC.

### 3.2. Biochemical Characterization of the Chon-ABC

#### 3.2.1. The Effects of Temperature, pH and Buffer Concentration on the Chon-ABC Activity

**Figure 2:**
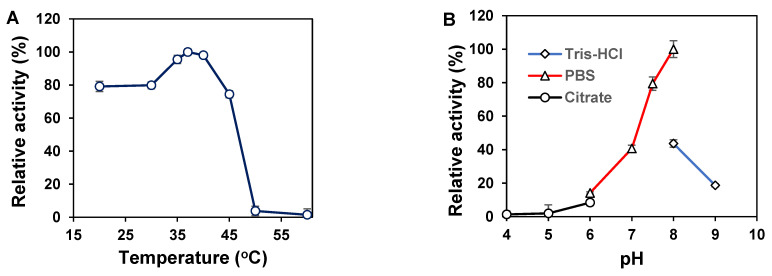

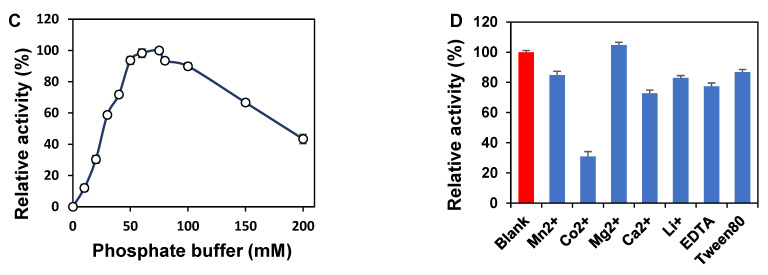
Biochemical characterization of the recombinant Chon-ABC. The effects of temperature (**A**), pH (**B**), PBS concentration (**C**), and metal ions and surfactant (**D**) on the enzyme activity at 37 °C.

The temperature dependence of the Chon-ABC activity was tested in a range of 20~60 °C. The results suggested that the activity of Chon-ABC increased with the increase of temperature, and it reached the maximum at 37 °C as shown in Figure 2A. The Chon-ABC retained 79–97% activity from 20 to 35 °C. It decreased sharply to 3% of the maximum activity when the temperature increased from 35 to 60 °C. The optimal pH for the Chon-ABC was found to be pH 8.0. It exhibited less than 40% relative activities when pH values were below 7.0 or above 8.0 (Figure 2B). The buffer concentration also had a strong influence on the enzyme activity and the maximal activity of the Chon-ABC was obtained in 75 mM PBS (Figure 2C). And the activity of the enzyme did display significant changes in a concentration range of 0–70 mM.

#### 3.2.2. The Effects of Metal Ions and Surfactants on the Chon-ABC Activity

The effects of metal ions and surfactants on the activities of the Chon-ABC were investigated, and the results (Figure 2D) indicate that there is no significant improvement in the presence of metal ions and surfactants, except for Mg^2+^ which slightly enhanced the Chon-ABC activity (104% relative activity). The inhibitory effects of 1 mM Mn^2+^, Co^2+^, Ca^2+^, Li^+^, EDTA, and 5% tween-80 were observed and retained 85%, 31%, 73%, 83%, 77%, and 87% relative activities compared with control, respectively.

#### 3.2.3. The Thermal and Storage Stability of the Chon-ABC

**Figure 3:**
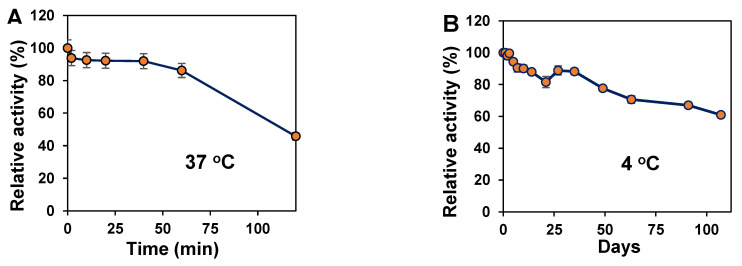
The stability of the recombinant Chon-ABC at 37 °C (**A**) and 4 °C (**B**) in 75 mM pH 8.0 PBS.

The thermostability of the enzyme was determined at 37 °C for 120 min. The results show that the half-life of the Chon-ABC is 127 min when incubated at 37 °C (Figure 3A). The storage stability was determined at 4 °C as well. The enzyme still retained 61% of initial activity after 107 days of storage (Figure 3B).

#### 3.2.4. Kinetic Parameters

**Figure 4:**
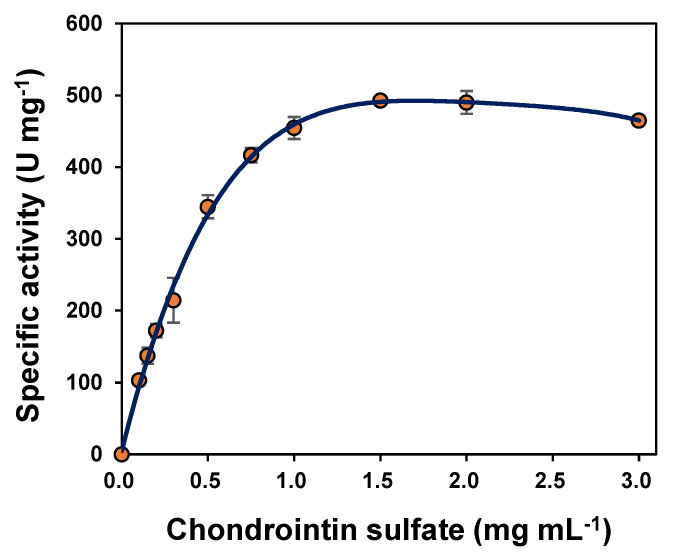
The effect of chondroitin sulfate concentration on the activity of the Chon-ABC at 37 °C in 75 mM pH 8.0 PBS.

The reaction rate was measured in pH 8.0 PBS. As shown in Figure 4, the kinetic parameters of the Chon-ABC were evaluated by changing the CS concentrations from 0 to 3 mg mL^−1^. After the nonlinear regression analysis, the *V_max_* and *K_m_* values of the Chon-ABC were 541.3 U mg^−1^ and 0.54 mg mL^−1^, respectively (Figure 4).

### 3.3. The Analysis of the CS and Hydrolyzed CS

#### 3.3.1. The GPC Analysis of the CS and Hydrolyzed CS

**Figure 5:**
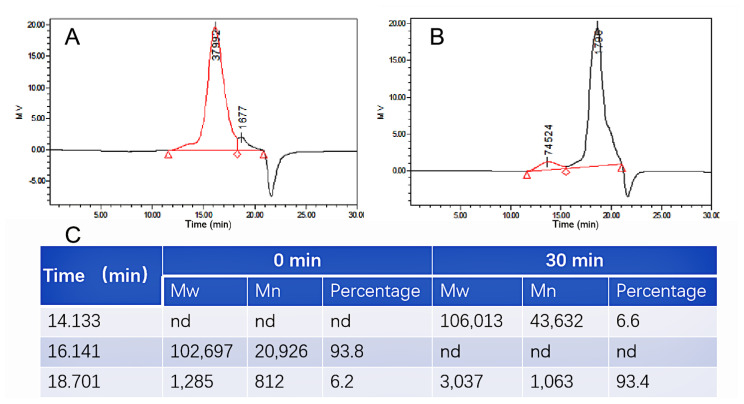
GPC analysis of the CS (**A**) and hydrolyzed CS (**B**) and the weight-average molecular weight (*M_w_*) and the distributions (**C**). The 0 min and 30 min in C are the reaction time. *M_n_* and nd mean the number-average molecular weight and not determined, respectively.

To evaluate the molecular weight and the distribution of the substrate and enzymatic depolymerized product, GPC was performed and shown in Figure 5. Before the reaction, the weight-average molecular weight (*M_w_*) of the substrate was 102,697 Da, which accounted for 93.8% of all components (Figure 5A,C). After 30 min enzymatic depolymerization, the *M_w_* decreased to 3037 Da (~93.4 % in all components) (Figure 5B,C). The conversion rate of substrate is up to 92.7%, suggesting that most of the CS was depolymerized into LMWCS.

#### 3.3.2. HPLC and MS Analysis of the CS and Hydrolyzed CS

**Figure 6:**
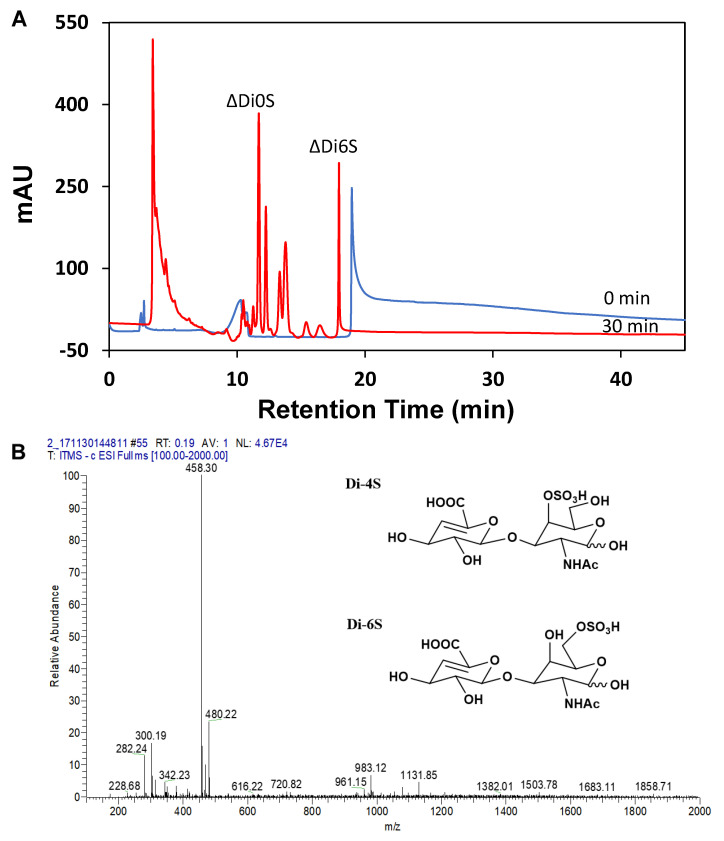
SAX-HPLC (**A**) and MS (**B**) analysis of the CS and hydrolyzed CS after 30 min enzymatic **reaction**. ΔDi0S = ΔUA-GalNAc; ΔDi6S = ΔUA-GalNAc6S.

The substrate and product were analyzed by SAX-HPLC and MS (Figure 6). The spectra of HPLC in Figure 6A show the components of the hydrolyzed CS after 30 min enzymatic depolymerization. In the spectra, the compound with retention time at around 13.8 min corresponded to the non-sulfated disaccharide, whereas the retention time at 17.9 min was attributed to the 6-sulfated disaccharide in the hydrolyzed CS [28]. The spectra of ESI-MS (Figure 6B) further supported the highly specific depolymerization of CS with the Chon-ABC. In the 30 min sample, the peak of *m/z* = 458 was attributed to only mono-sulfated unsaturated disaccharide, and its intensity suggested the degree of depolymerization of CS. For the 4S disaccharide, the peak of the characteristic product ion was *m/z* = 300, while that of the 6S disaccharide was *m/z* = 282.

#### 3.3.3. The NMR Analysis of the CS and Hydrolyzed CS

**Figure 7:**
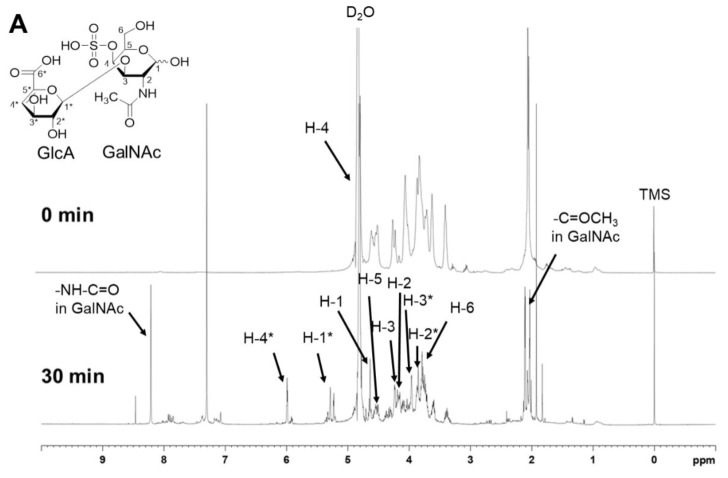

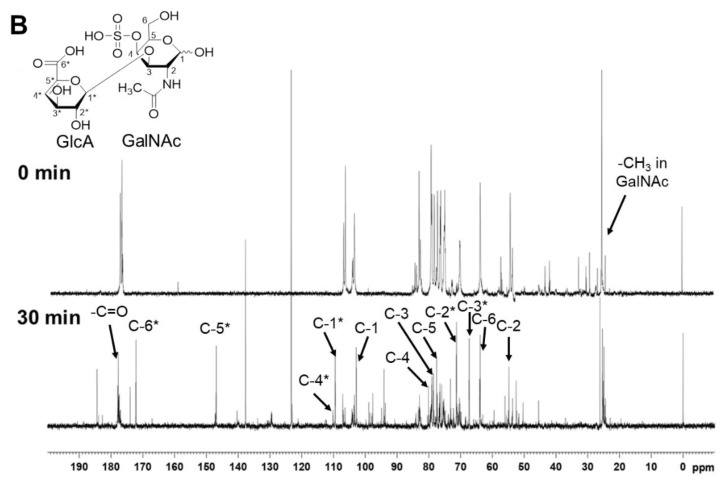
^1^H NMR (**A**) and ^13^C NMR (**B**) spectra of the CS and hydrolyzed CS. GlcA: glucuronic acid; GalNAc: N-acetylgalactosamine. The ^1^H NMR and ^13^C NMR spectra were obtained using an Agilent DD2 spectrometer at 25 °C, and the nuclear magnetic frequency was 600 MHz.

The ^1^H NMR and ^13^C NMR were performed to investigate the structural changes after enzymatic depolymerization. In Figure 7A of ^1^H NMR, the signals at 2.0 ppm can be assigned to the acetamido methyl of N-acetyl-D-galactosamine (GalNAc) [39]. In addition, the spectra presented broad-shaped signals located in the region between 4.8 and 3.0 ppm, which was consistent with the attribution of most hydrogen in disaccharides. They were attributed to the cross-ring protons which did not change in the CS and hydrolyzed CS. The chemical shift around 4.2 ppm was for H-2 and H-3 in GalNAc. The peak at 4.8 ppm was related to H-4 of GalNAc-4S, which overlapped with the signals of D_2_O. However, there were also some obvious changes after 30 min of depolymerization. The new signals at 5.3 ppm were assigned to the H-1 of glucuronic acid (GlcA), while the peak of 6.0 ppm referred to H-4 of GlcA. Moreover, the signal at 8.2 ppm was assigned to GalNAc-NHC=O, where the active hydrogen did not appear in the spectrum of the CS. Therefore, the same fine structure including the 4-sulfated group, β-1,3 glycosidic bonds, and carbonyl in the disaccharide of the CS and hydrolyzed CS was not changed after the enzymatic depolymerization.

Compared with ^1^H NMR, ^13^C NMR showed the information about carbon atoms. Figure 7B is the ^13^C NMR spectra of the substrate CS and hydrolyzed CS. At around 178 ppm, the signals of carbonyl (C=O) carbon in the GalNAc amide structure and carboxylic acid in the GlcA were found in the CS spectra. Meanwhile, the signals of C-6* (carbonyl carbon) in the GlcA were found at 172 ppm in the hydrolyzed CS, which may be caused by the π-π conjugation effect [40]. In addition, the signals at around 103 and 110 ppm were attributed to C-1 in the GalNAc and C-1* in the GlcA, and the signals at around 65 ppm referred to C-6 in GalNAc. The signals of C-2 from GalNAc were located at around 54 ppm. The signals between 60 and 80 ppm were derived from the other carbons in the disaccharide, while the signal at 20 ppm could be referred to the carbon atoms in -CH_3_ [40]. All the results show that the new unsaturated carbonyls were generated in the GlcA of hydrolyzed samples, further suggesting that the main enzymatic site was the β-1,4 glycosidic bonds between the disaccharides.

### 3.4. Molecular Docking of the Chon-ABC and Sulfated Glycosaminoglycan

**Figure 8:**
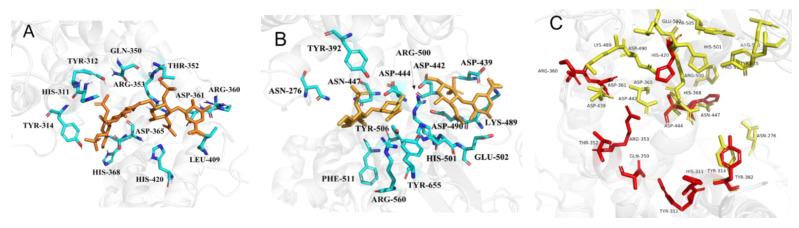
Visualization of molecular docking of the Chon-ABC and CS. The Chon-ABC and CS (**A**), the *pv*-cABC (**B**), and the superimposition of the Chon-ABC and *pv*-cABC (**C**). Molecular docking was performed with AutoDock4 (Lamarckian genetic algorithm, number of runs 100).

The chondroitinase ABC from *proteus vulgaris* (*pv*-cABC) was fully studied before, which has a high specific activity of 174.6 U mg^−1^ to CS, and the crystal structure and active site have already been identified [27]. Figure 8 depicts the results of molecular docking and superimposition of the Chon-ABC from *B. thetaiotaomicron*, as well as *pv*-cABC (PDB: 1HN0) with a sulfated glycosaminoglycan molecule. In Figure 8A, the results demonstrate that CS interacts with the Chon-ABC by several active residues (His-311, Tyr-312, Tyr-314, Gln-350, Thr-352, Arg-353, Arg-360, Asp-361, Asp-365, His-368, Leu-409, His-420). Different from the Chon-ABC, the visualization of the *pv*-cABC interaction with CS shows that the active sites include Asn-272, Tyr-392, Asp-439, Asp-442, Asp-444, Asn-447, Lys-489, Asp-490, Arg-500, His-501, Glu-502, Tyr-506, Phe-511, Arg-560, and Tyr-655 (Figure 8B). The free energy of the complex Chon-ABC-CS was −16.074 kcal mol^−1^, while the free energy of the complex *pv*-cABC-CS was −6.725 kcal mol^−1^. Figure 8C is the visualization of the superimposition between the Chon-ABC (red stick) and *pv*-cABC (yellow stick). The figure indicates that the Chon-ABC has a larger active pocket than that of *P. vulgaris*.

## 4. Discussion

LMWCS is an important compound used in medicine [2,3,4,5,6], food additives [7], and osteoarthritis [8]. The chemical manufacturing ways are still limited due to the expensive cost, toxic reagents, and low efficiency [14,15,41]. More importantly, the unwanted side reactions inevitably occurred in acidic, oxidative, or radiation processes which produce a complex mixture of modified LMWCS and further decrease the LMWCS biological activity and yield [18,42]. However, enzymatic preparation is an attractive method in which these problems could be avoided.

The advantages of the enzymatic method are that it is environment-friendly and efficient. In this work, a Chon-ABC was successfully cloned, expressed, and characterized. The results show that the Chon-ABC displayed a maximum activity of 500.4 U mg^−1^ in pH 8.0 PBS at 37 °C, which was 2.87-fold higher than that of pv-cABC [27]. The buffer concentration at 75 mM was friendly to the environment and convenient for extraction. Even though the Chon-ABC in this work belonged to the same subfamily of glycosaminoglycan lyase, its amino sequence has only 34% identity to the reported [27]. The Chon-ABC showed high storage stability with a half-life of 133 d at 4 °C which was conducive to subsequent research and industrial applications. The function or biochemical performance of the Chon-ABC could be further analyzed based on its primary sequence and steric structure. The molecular docking indicated that the active sites of the Chon-ABC have a smaller-sized side-chain of amino acid residues (Gln350, Thr352, Asp361, and Asp365) than that of the pv-cABC. It means that the enzyme has a larger active pocket, which can allow the substrate and product to enter or exclude easily during the catalysis. Moreover, the binding energies of the complex of Chon-ABC-CS and pv-cABC-CS were calculated by g_mmpbsa (http://rashmikumari.github.io/g_mmpbsa, accessed on 4 December 2021). The binding energy of Chon-ABC-CS (−347.1546 kcal mol^−1^) was lower than that of the pv-cABC-CS (−231.2428 kcal mol^−1^) which means that the Chon-ABC has a stronger affinity for CS during the enzymatic catalysis. It could also prove that the Chon-ABC has a higher activity theoretically. This would be more competitive in the enzymatic production of LMWCS.

The spectra of HPLC, MS, and GPC demonstrated that the main components of the hydrolyzed CS depolymerized by the Chon-ABC were 4-sulfated and 6-sulfated LMWCS. They are different from monosaccharides such as GalNAc and GlcA which had no biological activities such as neuronal development, coagulation, joint inflammation, cellular trafficking, and proliferation [3,4,5,6]. Moreover, the ratio of ions 282:300 can be used to calculate the amount of each component [43]. Here, the ratio of 4-sulfated and 6-sulfated disaccharide was 4:3 which agreed with the reports about the analysis of LMWCS [21,44]. The spectra of ^1^H and ^13^C NMR proved the formation of Δ4,5-unsaturated bond in the GlcA. It was also evident that the main product of depolymerization was the LMWCS consisting of GalNAc and GlcA. In addition, the key functional groups in the basic unit such as the 4-sulfated group in GalNAc, the amide in the GalNAc, and the carboxyl in the GlcA were well preserved in the enzymatic depolymerization. These groups, which played important roles in the biological activities [35], are easy to damage in chemical or physical methods. Thus, it was also indicated that the Chon-ABC used in this study could effectively degrade the CS into LMWCS without breaking the basic structure.

It is known that the biological activity of CS is highly dependent on the relative molecular mass (Mr) [1,45]. According to the previous report, when the depolymerized CS had the relative molecular weight of 500–5000 Da, it had superior bioactivity in relieving the pain and improving the functional status of osteoarthritis patients [40,46]. In the present work, the molecular weight of the produced LMWCS exactly falls in this range, as the Chon-ABC depolymerized more than 90% of CS (Mw: 102,697 Da) into LMWCS (Mw: 3037 Da) in 30 min. As expected, the LMWCS in this method has great bioactivity in the repair of osteoarthritis. The analysis of HPLC, MS, GPC, ^1^H NMR, and ^13^C NMR indicated that the Chon-ABC could specifically hydrolyze β-1,4 glycosidic bonds between the repeat disaccharides and it does not break β-1,3 glycosidic bonds in the GlcA and GalNAc. The main product of the enzymatic catalysis was LMWCS as the basic functional groups such as the 4/6-sulfated group in GalNAc, the amide in the GalNAc, and the carboxyl in the GlcA were well preserved. In summary, the Chon-ABC could be an ideal enzyme for the industrial enzymatic preparation of LMWCS.

## 5. Conclusions

In summary, a chondroitin sulfate lyase ABC (Chon-ABC) from *Bacteroides thetaiotaomicron* was successfully cloned and expressed heterologously in *E. coli*. The characterization showed it has a maximal specific activity of 500.4 U mg^−1^ in pH 8.0 PBS at 37 °C, and the *K_m_* and *V_max_* of the Chon-ABC were 0.54 mM and 541.3 U mg^−1^, respectively, which have never been reported before. In addition, its excellent stability provides the possibility for further research and industrial application. Enzymatic depolymerization of chondroitin sulfate with the Chon-ABC was performed at 37 °C for 30 min, and the substrate and product were analyzed by GPC, HPLC, MS, and NMR. The results reveal the molecular weight was decreased from 102,697 Da to 3037 Da by enzymatic depolymerization and the functional groups for biological activity including the 4/6-sulfated group in GalNAc, the amide in the GalNAc, and the carboxyl in the GlcA were well preserved. Therefore, the Chon-ABC could be an ideal catalyst for the industrial preparation of LMWCS.

## Data Availability

The data presented in this study are openly available in polymers.

## References

[1] Lauder R.M. (2009). Chondroitin Sulphate: A Complex Molecule with Potential Impacts on a Wide Range of Biological Systems. Complement. Ther. Med..

[2] Buyue Y., Sheehan J.P. (2009). Fucosylated Chondroitin Sulfate Inhibits Plasma Thrombin Generation via Targeting of the Factor IXa Heparin-Binding Exosite. Blood.

[3] He M., Wang J., Hu S., Wang Y., Xue C., Li H. (2014). The Effects of Fucosylated Chondroitin Sulfate Isolated from Isostichopus Badionotus on Antimetastatic Activity via Down-Regulation of Hif-1α and Hpa. Food Sci. Biotechnol..

[4] Li J., Li S., Yan L., Ding T., Linhardt R.J., Yu Y., Liu X., Liu D., Ye X., Chen S. (2017). Fucosylated Chondroitin Sulfate Oligosaccharides Exert Anticoagulant Activity by Targeting at Intrinsic Tenase Complex with Low FXII Activation: Importance of Sulfation Pattern and Molecular Size. Eur. J. Med. Chem..

[5] Wu N., Zhang Y., Ye X., Hu Y., Ding T., Chen S. (2016). Sulfation Pattern of Fucose Branches Affects the Anti-Hyperlipidemic Activities of Fucosylated Chondroitin Sulfate. Carbohydr. Polym..

[6] Zhao L., Lai S., Huang R., Wu M., Gao N., Xu L., Qin H., Peng W., Zhao J. (2013). Structure and Anticoagulant Activity of Fucosylated Glycosaminoglycan Degraded by Deaminative Cleavage. Carbohydr. Polym..

[7] Vázquez J.A., Blanco M., Fraguas J., Pastrana L., Pérez-Martín R. (2016). Optimisation of the Extraction and Purification of Chondroitin Sulphate from Head By-Products of Prionace Glauca by Environmental Friendly Processes. Food Chem..

[8] Michel B.A., Stucki G., Frey D., De Vathaire F., Vignon E., Bruehlmann P., Uebelhart D. (2005). Chondroitins 4 and 6 Sulfate in Osteoarthritis of the Knee: A Randomized, Controlled Trial. Arthritis Rheum..

[9] Du Souich P., Garcia A.G., Verges J., Montell E. (2009). Immunomodulatory and Anti-Inflammatory Effects of Chondroitin Sulphate. J. Cell. Mol. Med..

[10] Henrotin Y., Lambert C. (2013). Chondroitin and Glucosamine in the Management of Osteoarthritis: An Update. Curr. Rheumatol. Rep..

[11] Bakalash S., Rolls A., Lider O., Schwartz M. (2007). Chondroitin Sulfate-Derived Disaccharide Protects Retinal Cells from Elevated Intraocular Pressure in Aged and Immunocompromised Rats. Investig. Opthalmol. Vis. Sci..

[12] Ebert S., Schoeberl T., Walczak Y., Stoecker K., Stempfl T., Moehle C., Weber B.H.F., Langmann T. (2008). Chondroitin Sulfate Disaccharide Stimulates Microglia to Adopt a Novel Regulatory Phenotype. J. Leukoc. Biol..

[13] Ali S.S., Kenawy E.-R., Sonbol F.I., Sun J., Al-Etewy M., Ali A., Huizi L., El-Zawawy N.A. (2019). Pharmaceutical Potential of a Novel Chitosan Derivative Schiff Base with Special Reference to Antibacterial, Anti-Biofilm, Antioxidant, Anti-Inflammatory, Hemocompatibility and Cytotoxic Activities. Pharm. Res..

[14] Guo L.-B., Zhu C.-Y., Wu Y.-B., Fan X.-M., Zhang Y.-W. (2021). A Novel Chondroitin AC Lyase from Pedobacter Xixiisoli: Cloning, Expression, Characterization and the Application in the Preparation of Oligosaccharides. Enzym. Microb. Technol..

[15] Wu N., Ye X., Guo X., Liao N., Yin X., Hu Y., Sun Y., Liu D., Chen S. (2013). Depolymerization of Fucosylated Chondroitin Sulfate from Sea Cucumber, Pearsonothuria Graeffei, via 60Co Irradiation. Carbohydr. Polym..

[16] Lian C., Ruan L., Shang D., Wu Y., Lu Y., Lu P., Yang Y., Wei Y., Dong X., Ren D. (2016). Heparin-Binding Epidermal Growth Factor-Like Growth Factor as a Potent Target for Breast Cancer Therapy. Cancer Biother. Radiopharm..

[17] Liu C.-Y., Su W.-B., Guo L.-B., Zhang Y.-W. (2020). Cloning, Expression, and Characterization of a Novel Heparinase I from Bacteroides Eggerthii. Prep. Biochem. Biotechnol..

[18] Shively J.E., Conrad H.E. (1976). Nearest Neighbor Analysis of Heparin: Identification and Quantitation of the Products Formed by Selective Depolymerization Procedures. Biochemistry.

[19] Du F., Lou J., Jiang R., Fang Z., Zhao X., Niu Y., Zou S., Zhang M., Gong A., Wu C. (2017). Hyaluronic Acid-Functionalized Bismuth Oxide Nanoparticles for Computed Tomography Imaging-Guided Radiotherapy of Tumor. Int. J. Nanomed..

[20] Kang Z., Zhou Z., Wang Y., Huang H., Du G., Chen J. (2018). Bio-Based Strategies for Producing Glycosaminoglycans and Their Oligosaccharides. Trends Biotechnol..

[21] Takashima M., Watanabe I., Miyanaga A., Eguchi T. (2021). Substrate Specificity of Chondroitinase ABC I Based on Analyses of Biochemical Reactions and Crystal Structures in Complex with Disaccharides. Glycobiology.

[22] Imada K., Oka H., Kawasaki D., Miura N., Sato T., Ito A. (2010). Anti-arthritic action mechanisms of natural chondroitin sulfate in human articular chondrocytes and synovial fibroblasts. Biol. Pharm. Bull..

[23] Tinga Z., Yina F., Mao G., Feng W., Zou Y., Yec Z., Yang L., Wu X. (2017). Purification, Characterization and Antioxidant Activities of Enzymolysis Polysaccharide from Grifola Frondosa. Iran. J. Pharm. Res..

[24] Kasinathan N., Volety S.M., Josyula V.R. (2014). Chondroitinase: A Promising Therapeutic Enzyme. Crit. Rev. Microbiol..

[25] Chen Z., Li Y., Feng Y., Chen L., Yuan Q. (2015). Enzyme Activity Enhancement of Chondroitinase ABC I from *Proteus Vulgaris* by Site-Directed Mutagenesis. RSC Adv..

[26] Chen Z., Li Y., Yuan Q. (2015). Expression, Purification and Thermostability of MBP-Chondroitinase ABC I from *Proteus vulgaris*. Int. J. Biol. Macromol..

[27] Prabhakar V., Capila I., Bosques C.J., Pojasek K., Sasisekharan R. (2005). Chondroitinase ABC I from Proteus Vulgaris: Cloning, Recombinant Expression and Active Site Identification. Biochem. J..

[28] Shaya D., Hahn B.-S., Park N.Y., Sim J.-S., Kim Y.S., Cygler M. (2008). Characterization of Chondroitin Sulfate Lyase ABC from *Bacteroides Thetaiotaomicron* WAL2926. Biochemistry.

[29] Hong S.W., Kim B.T., Shin H.Y., Kim W.S., Lee K.S., Kim Y.S., Kim D.H. (2002). Purification and Characterization of Novel Chondroitin ABC and AC Lyases from *Bacteroides stercoris* HJ-15, a Human Intestinal Anaerobic Bacterium. Eur. J. Biochem..

[30] Zhu C., Zhang J., Zhang J., Jiang Y., Shen Z., Guan H., Jiang X. (2017). Purification and Characterization of Chondroitinase ABC from *Acinetobacter Sp.* C26. Int. J. Biol. Macromol..

[31] Bagherzadeh K., Maleki M., Golestani A., Khajeh K., Amanlou M. (2018). Chondroitinase ABC I Thermal Stability Is Enhanced by Site-Directed Mutagenesis: A Molecular Dynamic Simulations Approach. J. Biomol. Struct. Dyn..

[32] Gao L., Li Q., Deng Z., Brady B., Xia N., Zhou Y., Shi H. (2017). Highly Sensitive Protein Detection via Covalently Linked Aptamer to MoS2 and Exonuclease-Assisted Amplification Strategy. Int. J. Nanomed..

[33] Qian W., Wang Y., Zhu J., Mao C., Wang Q., Huan F., Cheng J., Liu Y., Wang J., Xiao H. (2015). The Toxic Effects of Bisphenol A on the Mouse Spermatocyte GC-2 Cell Line: The Role of the Ca^2+^-Calmodulin-Ca^2+^/Calmodulin-Dependent Protein Kinase II Axis. J. Appl. Toxicol..

[34] Li Y., Zhou Z., Chen Z. (2018). High-Level Production of ChSase ABC I by Co-Expressing Molecular Chaperones in Escherichia Coli. Int. J. Biol. Macromol..

[35] Yamagata T., Saito H., Habuchi O., Suzuki S. (1968). Purification and Properties of Bacterial Chondroitinases and Chondrosulfatases. J. Biol. Chem..

[36] Bougatef H., Krichen F., Capitani F., Amor I.B., Maccari F., Mantovani V., Galeotti F., Volpi N., Bougatef A., Sila A. (2018). Chondroitin Sulfate/Dermatan Sulfate from Corb (Sciaena Umbra) Skin: Purification, Structural Analysis and Anticoagulant Effect. Carbohydr. Polym..

[37] Morris G.M., Huey R., Lindstrom W., Sanner M.F., Belew R.K., Goodsell D.S., Olson A.J. (2009). AutoDock4 and AutoDockTools4: Automated Docking with Selective Receptor Flexibility. J. Comput. Chem..

[38] Berendsen H.J.C., Spoel D., Drunen R. (1995). GROMACS: A message-passing parallel molecular dynamics implementation. Comput. Phys. Commun..

[39] Rani A., Goyal A. (2016). A New Member of Family 8 Polysaccharide Lyase Chondroitin AC Lyase (Ps PL8A) from *Pedobacter Saltans* Displays Endo- and Exo-Lytic Catalysis. J. Mol. Catal. B Enzym..

[40] Lan R., Li Y., Shen R., Yu R., Jing L., Guo S. (2020). Preparation of Low-Molecular-Weight Chondroitin Sulfates by Complex Enzyme Hydrolysis and Their Antioxidant Activities. Carbohydr. Polym..

[41] Wu Z., Song H., Cui X., Pi C., Du W., Wu Y. (2013). Sulfonylation of Quinoline n -Oxides with Aryl Sulfonyl Chlorides via Copper-Catalyzed C–H Bonds Activation. Org. Lett..

[42] Hook M., Thunberg L., Fransson L.-A., Linker A. (1979). Structure of the Antithrombin-Binding Site in Heparin. Proc. Natl. Acad. Sci. USA.

[43] Zaia J. (2005). Principles of Mass Spectrometry of Glycosaminoglycans. J. Biomacromol. Mass Spectrom..

[44] Yamada S., Yoshida K., Sugiura M., Sugahara K. (1992). One- and Two-Dimensional ^1^H-NMR Characterization of Two Series of Sulfated Disaccharides Prepared from Chondroitin Sulfate and Heparan Sulfate/Heparin by Bacterial Eliminase Digestion. J. Biochem..

[45] Volpi N. (2004). Disaccharide Mapping of Chondroitin Sulfate of Different Origins by High-Performance Capillary Electrophoresis and High-Performance Liquid Chromatography. Carbohydr. Polym..

[46] Wang J., Zhang L., Jin Z. (2016). Separation and Purification of Low-Molecular-Weight Chondroitin Sulfates and Their Anti-Oxidant Properties. Bangladesh J. Pharmacol..

